# 2-Amino-6-nitro-1,3-benzothia­zol-3-ium hydrogen sulfate

**DOI:** 10.1107/S1600536811027620

**Published:** 2011-07-16

**Authors:** Hui-Fen Qian, Wei Huang

**Affiliations:** a College of Sciences, Nanjing University of Technology, Nanjing 210009, People’s Republic of China; b State Key Laboratory of Coordination Chemistry, Nanjing National Laboratory of Microstructures, School of Chemistry and Chemical Engineering, Nanjing University, Nanjing 210093, People’s Republic of China

## Abstract

In the title molecular salt, C_7_H_6_N_3_O_2_S^+^·HSO_4_^−^, the 2-amino-6-nitro-1,3-benzothia­zole ring system is essentially planar [mean deviation = 0.0605 (4) Å]. In the crystal, N—H⋯O and O—H⋯O hydrogen-bonding inter­actions result in a layer motif.

## Related literature

For related compounds, see Glidewell *et al.* (2001 ▶); Lynch (2002 ▶); Lynch & Duckhouse (2001 ▶); You *et al.* (2009 ▶).
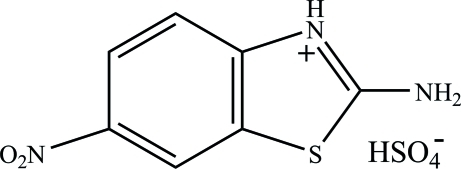


## Experimental

### 

#### Crystal data

C_7_H_6_N_3_O_2_S^+^·HSO_4_^−^
*M* *_r_* = 293.28
Monoclinic, 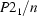

*a* = 7.849 (6) Å
*b* = 16.219 (12) Å
*c* = 9.191 (7) Å
β = 108.584 (10)°
*V* = 1109.0 (14) Å^3^
*Z* = 4
Mo *K*α radiation
μ = 0.51 mm^−1^
*T* = 291 K
0.16 × 0.14 × 0.12 mm

#### Data collection

Bruker 1K CCD area-detector diffractometer
Absorption correction: multi-scan (*SADABS*; Bruker, 2000 ▶) *T*_min_ = 0.923, *T*_max_ = 0.942
5436 measured reflections
1958 independent reflections
1586 reflections with *I* > 2σ(*I*)
*R*_int_ = 0.097

#### Refinement

*R*[*F* ^2^ > 2σ(*F* ^2^)] = 0.061
*wR*(*F* ^2^) = 0.159
*S* = 0.98
1958 reflections
163 parameters
H-atom parameters constrained
Δρ_max_ = 0.52 e Å^−3^
Δρ_min_ = −0.60 e Å^−3^

### 

Data collection: *SMART* (Bruker, 2000 ▶); cell refinement: *SAINT* (Bruker, 2000 ▶); data reduction: *SAINT*; program(s) used to solve structure: *SHELXTL* (Sheldrick, 2008 ▶); program(s) used to refine structure: *SHELXTL*; molecular graphics: *SHELXTL*; software used to prepare material for publication: *SHELXTL*.

## Supplementary Material

Crystal structure: contains datablock(s) global, I. DOI: 10.1107/S1600536811027620/ff2020sup1.cif

Structure factors: contains datablock(s) I. DOI: 10.1107/S1600536811027620/ff2020Isup2.hkl

Supplementary material file. DOI: 10.1107/S1600536811027620/ff2020Isup3.cml

Additional supplementary materials:  crystallographic information; 3D view; checkCIF report

## Figures and Tables

**Table 1:** Hydrogen-bond geometry (Å, °)

*D*—H⋯*A*	*D*—H	H⋯*A*	*D*⋯*A*	*D*—H⋯*A*
N1—H1*A*⋯O4^i^	0.86	1.97	2.825 (4)	171
N2—H2*A*⋯O6^i^	0.86	2.02	2.867 (4)	170
N2—H2*B*⋯O6^ii^	0.86	2.10	2.888 (4)	151
O3—H3*A*⋯O4^iii^	0.82	1.86	2.664 (4)	166

Symmetry codes: (i) 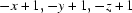; (ii) 
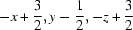
; (iii) 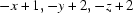.

**Fig. 1.**
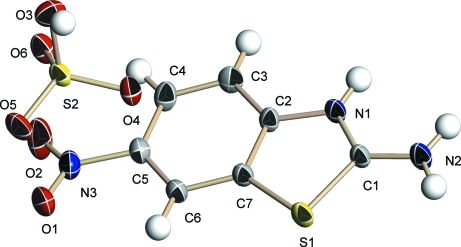
An ORTEP drawing of the title compound with the atom-numbering scheme. Displacement ellipsoids are drawn at the 30% probability level.

## supplementary crystallographic information

### Comment

There have been three single-crystal structural investigations on 2-amino-6-nitro-1,3-benzothiazole, namely 2-amino-6-nitro-1,3-benzothiazole (Glidewell *et al.*, 2001),its monohydrate (Lynch *et al.*, 2002) and its PtCl_2_ complex (Lynch & Duckhouse, 2001). We have previously reported the single-crystal structure of 2-aminobenzimidazolium hydrogen sulfate (You *et al.*, 2009). In this work, we describe the single-crystal structure of a hydrogen sulfate salt of 2-amino-6-nitro-1,3-benzothiazole.

The atom-numbering scheme of the title compound is shown in Fig. 1, while selected bond distances and bond angles are given in Table 1. The 2-amino-6-nitro-1,3-benzothiazole skeleton of the title compound is is essentially planar with the mean deviation of 0.0605 (4) Å. The proton is delocalized within the thiozole ring although it is added to the nitrogen atom. With regard to the hydrogen sulfate anion, the hydrogen atom is added to the O3 atom of SO_4_ group due to the obviously longer O3–S2 bond length. In the crystal packing, N—H···O and O—H···O hydrogen-bond interactions are found between adjacent molecules giving rise to a layer motif.

### Experimental

The treatment of 2-amino-6-nitro-1,3-benzothiazole dissolved in methanol with an excess of sulfuric acid yields the title compound. Single crystals suitable for X-ray diffraction measurement were obtained after 7 days' slow evaporation of the mother liquid at room temperature in air. Anal. Calcd. For C_7_H_6_N_3_O_2_S^+^.HSO_4_^-^: C, 28.67; H, 2.41; N, 14.33%. Found: C, 28.53; H, 2.66; N, 14.44%.

### Refinement

The non-hydrogen atoms were refined anisotropically, whereas the H atoms bonded with carbon, nitrogen and oxygen atoms were placed in geometrically idealized positions (C—H = 0.93 Å, N—H = 0.86 Å and O—H = 0.82 Å) and refined as riding atoms, with *U*_iso_(H) = 1.2*U*_eq_(C) and 1.2*U*~eq~(N) and *U*ĩso~(H) = 1.5*U*~eq~(O).

### Crystal data

**Table:**

C_7_H_6_N_3_O_2_S^+^·HSO_4_^−^	*F*(000) = 600
*M**_r_* = 293.28	*D*_x_ = 1.757 Mg m^−^^3^
Monoclinic, *P*2_1_/*n*	Mo *K*α radiation, λ = 0.71073 Å
Hall symbol: -P 2yn	Cell parameters from 2618 reflections
*a* = 7.849 (6) Å	θ = 2.3–28.0°
*b* = 16.219 (12) Å	µ = 0.51 mm^−^^1^
*c* = 9.191 (7) Å	*T* = 291 K
β = 108.584 (10)°	Block, colourless
*V* = 1109.0 (14) Å^3^	0.16 × 0.14 × 0.12 mm
*Z* = 4	

### Data collection

**Table:**

Bruker 1K CCD area-detector diffractometer	1958 independent reflections
Radiation source: fine-focus sealed tube	1586 reflections with *I* > 2σ(*I*)
graphite	*R*_int_ = 0.097
φ and ω scans	θ_max_ = 25.0°, θ_min_ = 2.5°
Absorption correction: multi-scan (*SADABS*; Bruker, 2000)	*h* = −9→6
*T*_min_ = 0.923, *T*_max_ = 0.942	*k* = −19→18
5436 measured reflections	*l* = −8→10

### Refinement

**Table:**

Refinement on *F*^2^	Primary atom site location: structure-invariant direct methods
Least-squares matrix: full	Secondary atom site location: difference Fourier map
*R*[*F*^2^ > 2σ(*F*^2^)] = 0.061	Hydrogen site location: inferred from neighbouring sites
*wR*(*F*^2^) = 0.159	H-atom parameters constrained
*S* = 0.98	*w* = 1/[σ^2^(*F*_o_^2^) + (0.1126*P*)^2^] where *P* = (*F*_o_^2^ + 2*F*_c_^2^)/3
1958 reflections	(Δ/σ)_max_ < 0.001
163 parameters	Δρ_max_ = 0.52 e Å^−^^3^
0 restraints	Δρ_min_ = −0.60 e Å^−^^3^

### Special details

**Table:**

Experimental. The structure was solved by direct methods (Bruker, 2000) and successive difference Fourier syntheses.
Geometry. All e.s.d.'s (except the e.s.d. in the dihedral angle between two l.s. planes) are estimated using the full covariance matrix. The cell e.s.d.'s are taken into account individually in the estimation of e.s.d.'s in distances, angles and torsion angles; correlations between e.s.d.'s in cell parameters are only used when they are defined by crystal symmetry. An approximate (isotropic) treatment of cell e.s.d.'s is used for estimating e.s.d.'s involving l.s. planes.
Refinement. Refinement of *F*^2^ against ALL reflections. The weighted *R*-factor *wR* and goodness of fit *S* are based on *F*^2^, conventional *R*-factors *R* are based on *F*, with *F* set to zero for negative *F*^2^. The threshold expression of *F*^2^ > σ(*F*^2^) is used only for calculating *R*-factors(gt) *etc*. and is not relevant to the choice of reflections for refinement. *R*-factors based on *F*^2^ are statistically about twice as large as those based on *F*, and *R*- factors based on ALL data will be even larger.

### Fractional atomic coordinates and isotropic or equivalent isotropic displacement parameters (Å^2^)

**Table:**

	*x*	*y*	*z*	*U*_iso_*/*U*_eq_	
C1	0.7190 (4)	0.12918 (17)	0.4541 (3)	0.0400 (7)	
C2	0.7416 (4)	−0.00938 (16)	0.5027 (3)	0.0379 (7)	
C3	0.7086 (5)	−0.09352 (18)	0.4792 (4)	0.0483 (8)	
H3	0.6294	−0.1131	0.3874	0.058*	
C4	0.7959 (5)	−0.14661 (19)	0.5950 (4)	0.0501 (8)	
H4	0.7790	−0.2032	0.5815	0.060*	
C5	0.9089 (4)	−0.11571 (17)	0.7315 (4)	0.0427 (7)	
C6	0.9441 (4)	−0.03251 (17)	0.7588 (3)	0.0427 (7)	
H6	1.0207	−0.0132	0.8520	0.051*	
C7	0.8588 (4)	0.02045 (17)	0.6393 (3)	0.0387 (7)	
N1	0.6668 (3)	0.05352 (15)	0.4010 (3)	0.0412 (6)	
H1A	0.5927	0.0447	0.3107	0.049*	
N2	0.6630 (4)	0.19655 (15)	0.3774 (3)	0.0524 (8)	
H2A	0.5874	0.1941	0.2861	0.063*	
H2B	0.7014	0.2436	0.4178	0.063*	
N3	0.9911 (4)	−0.17264 (18)	0.8562 (3)	0.0538 (7)	
O1	1.0531 (3)	−0.14566 (16)	0.9864 (3)	0.0651 (7)	
O2	0.9914 (5)	−0.24573 (18)	0.8252 (3)	0.0885 (10)	
O3	0.4172 (3)	0.88694 (13)	1.0011 (3)	0.0602 (7)	
H3A	0.3934	0.9305	1.0357	0.090*	
O4	0.6040 (3)	0.96348 (13)	0.8861 (2)	0.0479 (6)	
O5	0.7360 (3)	0.90117 (14)	1.1335 (3)	0.0607 (7)	
O6	0.6226 (3)	0.81597 (13)	0.9086 (3)	0.0549 (7)	
S1	0.87176 (11)	0.12757 (5)	0.63686 (9)	0.0506 (3)	
S2	0.60859 (9)	0.89227 (4)	0.98544 (8)	0.0401 (3)	

### Atomic displacement parameters (Å^2^)

**Table:**

	*U*^11^	*U*^22^	*U*^33^	*U*^12^	*U*^13^	*U*^23^
C1	0.0477 (16)	0.0359 (15)	0.0280 (16)	−0.0021 (12)	0.0003 (13)	0.0006 (12)
C2	0.0509 (16)	0.0330 (14)	0.0274 (15)	−0.0023 (12)	0.0092 (13)	0.0000 (11)
C3	0.067 (2)	0.0398 (16)	0.0321 (16)	−0.0075 (15)	0.0077 (14)	−0.0101 (13)
C4	0.072 (2)	0.0347 (15)	0.0430 (19)	0.0010 (15)	0.0172 (16)	0.0014 (14)
C5	0.0461 (16)	0.0434 (17)	0.0365 (17)	0.0047 (13)	0.0103 (14)	0.0073 (13)
C6	0.0451 (16)	0.0452 (16)	0.0315 (16)	−0.0033 (13)	0.0034 (13)	0.0035 (13)
C7	0.0449 (15)	0.0351 (15)	0.0312 (15)	−0.0024 (12)	0.0053 (12)	−0.0006 (11)
N1	0.0530 (14)	0.0383 (13)	0.0239 (12)	−0.0053 (11)	0.0005 (10)	−0.0023 (10)
N2	0.0697 (18)	0.0341 (13)	0.0363 (15)	−0.0036 (12)	−0.0073 (13)	−0.0003 (11)
N3	0.0579 (16)	0.0541 (17)	0.0479 (19)	0.0046 (13)	0.0149 (14)	0.0168 (14)
O1	0.0650 (15)	0.0766 (18)	0.0433 (16)	−0.0019 (13)	0.0025 (12)	0.0191 (13)
O2	0.131 (3)	0.0496 (15)	0.073 (2)	0.0216 (16)	0.0161 (17)	0.0176 (14)
O3	0.0525 (14)	0.0479 (13)	0.081 (2)	−0.0029 (10)	0.0230 (14)	−0.0050 (12)
O4	0.0656 (14)	0.0382 (11)	0.0354 (12)	0.0087 (10)	0.0099 (10)	0.0037 (9)
O5	0.0633 (15)	0.0678 (16)	0.0346 (13)	−0.0024 (11)	−0.0077 (11)	−0.0017 (11)
O6	0.0696 (15)	0.0379 (12)	0.0450 (14)	0.0118 (10)	0.0013 (11)	−0.0044 (10)
S1	0.0641 (6)	0.0364 (5)	0.0329 (5)	−0.0078 (3)	−0.0104 (4)	−0.0014 (3)
S2	0.0452 (5)	0.0362 (5)	0.0297 (5)	0.0029 (3)	−0.0010 (4)	−0.0023 (3)

### Geometric parameters (Å, °)

**Table:**

C1—N2	1.299 (4)	C6—H6	0.9300
C1—N1	1.336 (3)	C7—S1	1.741 (3)
C1—S1	1.725 (3)	N1—H1A	0.8600
C2—N1	1.382 (3)	N2—H2A	0.8600
C2—C7	1.386 (4)	N2—H2B	0.8600
C2—C3	1.393 (4)	N3—O1	1.220 (4)
C3—C4	1.370 (4)	N3—O2	1.220 (4)
C3—H3	0.9300	O3—S2	1.557 (3)
C4—C5	1.380 (5)	O3—H3A	0.8200
C4—H4	0.9300	O4—S2	1.466 (2)
C5—C6	1.384 (4)	O5—S2	1.416 (2)
C5—N3	1.453 (4)	O6—S2	1.446 (2)
C6—C7	1.387 (4)		
			
N2—C1—N1	124.2 (3)	C2—C7—S1	111.2 (2)
N2—C1—S1	123.5 (2)	C6—C7—S1	127.8 (2)
N1—C1—S1	112.3 (2)	C1—N1—C2	114.6 (2)
N1—C2—C7	111.8 (2)	C1—N1—H1A	122.7
N1—C2—C3	126.9 (3)	C2—N1—H1A	122.7
C7—C2—C3	121.3 (3)	C1—N2—H2A	120.0
C4—C3—C2	118.2 (3)	C1—N2—H2B	120.0
C4—C3—H3	120.9	H2A—N2—H2B	120.0
C2—C3—H3	120.9	O1—N3—O2	123.3 (3)
C3—C4—C5	119.7 (3)	O1—N3—C5	118.9 (3)
C3—C4—H4	120.2	O2—N3—C5	117.8 (3)
C5—C4—H4	120.2	S2—O3—H3A	109.5
C4—C5—C6	123.5 (3)	C1—S1—C7	90.14 (13)
C4—C5—N3	118.8 (3)	O5—S2—O6	114.58 (14)
C6—C5—N3	117.6 (3)	O5—S2—O4	112.84 (14)
C5—C6—C7	116.2 (3)	O6—S2—O4	111.16 (15)
C5—C6—H6	121.9	O5—S2—O3	108.89 (16)
C7—C6—H6	121.9	O6—S2—O3	102.90 (14)
C2—C7—C6	121.0 (3)	O4—S2—O3	105.57 (13)

### Hydrogen-bond geometry (Å, °)

**Table:**

*D*—H···*A*	*D*—H	H···*A*	*D*···*A*	*D*—H···*A*
N1—H1A···O4^i^	0.86	1.97	2.825 (4)	171
N2—H2A···O6^i^	0.86	2.02	2.867 (4)	170
N2—H2B···O6^ii^	0.86	2.10	2.888 (4)	151
O3—H3A···O4^iii^	0.82	1.86	2.664 (4)	166

Symmetry codes: (i) −*x*+1, −*y*+1, −*z*+1; (ii) −*x*+3/2, *y*−1/2, −*z*+3/2; (iii) −*x*+1, −*y*+2, −*z*+2.
